# From trash to treasure: detecting unexpected contamination in unmapped NGS data

**DOI:** 10.1186/s12859-019-2684-x

**Published:** 2019-04-18

**Authors:** Mara Sangiovanni, Ilaria Granata, Amarinder Singh Thind, Mario Rosario Guarracino

**Affiliations:** 1 Stazione Zoologica Anton Dohrn, Villa Comunale, Napoli, 80121 Italy; 2 High Performance Computing and Networking Institute, National Research Council of Italy, Via P. Castellino, 111, Napoli, 80131 Italy

**Keywords:** Contamination, Next generation sequencing, Unmapped reads

## Abstract

**Background:**

Next Generation Sequencing (NGS) experiments produce millions of short sequences that, mapped to a reference genome, provide biological insights at genomic, transcriptomic and epigenomic level. Typically the amount of reads that correctly maps to the reference genome ranges between 70% and 90%, leaving in some cases a consistent fraction of unmapped sequences. This ’misalignment’ can be ascribed to low quality bases or sequence differences between the sample reads and the reference genome. Investigating the source of the unmapped reads is definitely important to better assess the quality of the whole experiment and to check for possible downstream or upstream ’contamination’ from exogenous nucleic acids.

**Results:**

Here we propose DecontaMiner, a tool to unravel the presence of contaminating sequences among the unmapped reads. It uses a subtraction approach to identify bacteria, fungi and viruses genome contamination. DecontaMiner generates several output files to track all the processed reads, and to provide a complete report of their characteristics. The good quality matches on microorganism genomes are counted and compared among samples. DecontaMiner builds an offline HTML page containing summary statistics and plots. The latter are obtained using the state-of-the-art D3 javascript libraries. DecontaMiner has been mainly used to detect contamination in human RNA-Seq data. The software is freely available at http://www-labgtp.na.icar.cnr.it/decontaminer.

**Conclusions:**

DecontaMiner is a tool designed and developed to investigate the presence of contaminating sequences in unmapped NGS data. It can suggest the presence of contaminating organisms in sequenced samples, that might derive either from laboratory contamination or from their biological source, and in both cases can be considered as worthy of further investigation and experimental validation. The novelty of DecontaMiner is mainly represented by its easy integration with the standard procedures of NGS data analysis, while providing a complete, reliable, and automatic pipeline.

**Electronic supplementary material:**

The online version of this article (10.1186/s12859-019-2684-x) contains supplementary material, which is available to authorized users.

## Background

Standard NGS data analysis procedures involve a pre-processing step of quality assessment of the reads, followed by the alignment of the filtered ones to a reference genome. The mapped sequences are then investigated to extract the relevant biological information, such as transcripts expression, splicing events, nucleotide or structural variations and enriched regions of specific binding sites. Typically, the amount of reads that correctly maps to the specific reference genome ranges between 70% and 90%, leaving in some cases a consistent fraction of unmapped sequences.

The alignment process usually rejects two classes of reads: those which map several times along the genome (known as *multimapped reads*) and those which fail to correctly map on the reference. The first case is mostly due to the presence of repetitive elements, whereas the latter can be ascribed either to technical errors of the sequencing experiment, not detected or resolved through the quality assessment step, or to sequence differences between the reads and the reference. Investigating the reasons for this discrepancy may provide relevant information about the source of the so called *unmapped reads*.

As demonstrated by the literature, is not unusual that genetic material of microorganisms is present in biological samples undergoing sequencing [1–3]. The interest in detecting microorganisms-derived sequences in high-throughput data has grown up together with the knowledge that commensal and pathogenic microbes play an essential role in human health [4], thus fostering the possibility to find new disease-associated pathogens. Indeed, it is well established that the interplay of genetic and environmental factors determines the onset and progression of chronic diseases [5–8]. While the study of the genes and their tight regulation is a topic under constant investigation, the nature of the environmental components, their interaction with the genome and their specific role in physio-pathological mechanisms still represent a challenge of biological research.

Several studies have contributed to the definition of microbial populations in the human body as an important environmental factor, able to regulate the cellular behaviour and to influence the pathological processes [9–12]. These studies mainly focused on the gut microbiome characterisation, for which the regulatory function is very well known. Indeed, diseases as diabetes [13], coeliac disease [14, 15], obesity [16] and colorectal cancer [17] have been associated to the variation of the gut microbiome composition. The advent of high-throughput technologies allowed to understand that also other body sites, always considered sterile, such as lung, stomach and breast, host peculiar indigenous microbial populations [18–20]. Commensal microorganisms mostly show beneficial properties, especially in immune system homeostasis, but in particular conditions or predispositions can represent risk factors and are then defined as ’pathobionts’. The mechanisms by which these microorganisms are responsible for the onset of some chronic diseases are still unknown, although several studies have characterised a tight communication with host cells and identified the induction of DNA damage, chromosome instability and aneuploidy [21].

In literature, there are many shreds of evidence of the presence of contaminating organisms in high throughput sequencing data. The exogenous sequences can derive from the normal or altered tissues microbiome (upstream contamination) or environmental contamination during the samples processing (downstream contamination). Upstream contamination has been reported by several research groups which have used NGS techniques purposely to discover exogenous agents in human tissues samples and cell lines [22–25].

The detection of downstream contamination is equally important, since it can help to check the quality of the working environment and procedures. Strong et al. identified bacterial RNA, belonging to different taxa, in cell line data of different sequencing experiments. Microbial-derived sequences were present in polyA enriched RNA-Seq data and this finding made authors hypothesize that the exogenous reads did not derive from the specimens themselves but from downstream contamination [1]. Indeed, since bacteria are poorly polyadenylated [26], the mRNA enrichment step should remove eventual upstream contamination. Interestingly, laboratory-peculiar contamination has been found by a study which illustrated how various sequencing centres had specific signatures of contaminating genomes as ’time stamps’ [27]. Among the different NGS approaches, chromatin immunoprecipitation experiments (ChIP-Seq) are particularly characterised by a low read mappability, having very often a large portion (20-90%) of unaligned reads. Unmapped ChIP-Seq reads from *A. thaliana*, *Z. mays*, *H. sapiens*, and *D. Melanogaster* datasets were investigated and found contaminated by foreign sequences. The authors characterised the contaminant organisms and calculated the relative abundance for each dataset by taxonomic classification [28]. Quality assessment of the working environment is crucially important, since sequence-based methods are particularly sensitive to reagents and laboratory contamination. Mycoplasma contamination, which is particularly worth of attention for biologists, was searched in DNA sequences obtained from The Thousand Genome Project [29] and was detected in 7% of samples [30, 31]. Negative control libraries are strongly recommended to check contaminant DNAs in the context of high throughput sequencing, although they have a limited ability to recover low-frequency contaminants [32]. Low-abundance microbes and novel sequences are often hidden by common contaminants of NGS experiments, but their detection and characterization can be pursued by a detailed analysis of the unmapped reads [33]. Besides the contamination within samples, another alarm is represented by the cross-contamination among samples, that can invalidate the whole experimental protocol as well. Ballenghien et al. very recently highlighted the importance of examining NGS datasets for contamination and identifying the most susceptible steps, to propose targeted solutions. They uncovered indirect evidence that the vast majority of cross-contamination events is ascribable to sequencing centres [34].

Several tools, based on different computational approaches, have been developed and used for the detection of pathogens in high-throughput sequencing data. As far as we know, many of the available tools, such as PathSeq [35], SURPI [36] and RNA-COMPASS [37], are primarily aimed to the analysis of metagenomic data. Consequently, their pipelines are not appropriate for the detection of contamination among the unmapped reads. Moreover, they have features that might prevent their easy inclusion in an already established NGS analysis pipeline: PathSeq, for example, requires a commercial computing platform (i.e. Amazon Elastic Compute Cloud, EC2). SURPI aims at detecting microorganisms in complex clinical metagenomic samples, and, to this extent, it uses the entire NCBI nt and/or NCBI nr protein databases in comprehensive mode, requiring up to 2 terabytes of free space for the reference data creation. RNA Compass is specially designed for the simultaneous analysis of transcriptome and metatranscriptome data. It offers automation of analysis and works on the cloud and local servers but it requires a cumbersome installation. Other tools are DeconSeq [38], that works only on longer-read metagenomic datasets (> 150 bp mean read length), or CaPSID [39]. However, in order to reduce the required time and computational efforts, CaPSID works on BAM files provided by the user, who should take care of aligning the sequences both to human and to each pathogen reference genome of interest. Approaches developed to clean the fastq sequences from contaminants often require an a-priori knowledge, as expected in FastQ Screen [40] or contamination_screen [41], where the user must provide the genome of each putative contaminating species. Other tools, such as TruePure [42], have limits on the input size and process only small subsets of a fastq file.

Here we propose DecontaMiner, a tool developed to unravel the presence of contaminating sequences among the reads that fail to map to the reference genome. We described the first DecontaMiner prototype and analyzed the results in a previous work [43]. Here we present a more complete and mature version of the pipeline: the DecontaMiner’s code was completely reorganized in a way that permits to run all the processing steps separately. This is an essential feature, since it lets the user independently tune the parameters on the various analyzed databases as well as filter the results on different thresholds. Moreover, the code has been freely released for the first time and a companion website is provided, that permits an interactive visualization of the results. Unlike the above-cited tools, DecontaMiner has been conceived purposely to provide a method for investigating the possible foreign source of the unmapped reads. It does not require commercial platform or complicated installation, since it exploits several tools that are widely used by the sequencing community. It does not have limits of reads length and performs the alignment to several microorganism databases. It uses a subtraction approach in which the sequences are first filtered accordingly to quality parameters and then sequentially mapped to ribosomal, mitochondrial and foreign organisms databases. Although the experimental protocols provide a rRNA removal step, often this procedure is not sufficient, due to the high number of rRNA copies. The reads that do not map on human genome are then mapped, through a local alignment algorithm (MegaBLAST), to bacteria, fungi and viral genomes. DecontaMiner generates several output files to track all the processed reads, and to provide a complete report of their characteristics. The good quality matches on microorganism genomes are counted and compared among samples. Results are also generated as an offline HTML page, containing interactive plots. Furthermore, DecontaMiner provides an online page where the user can upload the result files and, setting the desired thresholds both on samples and detected contaminants, narrow the search and view the aggregated results in different charts. It is worth noting that, apart from being a tool for examining the source of unmapped reads, DecontaMiner can be also used as a pre-filtering step, i.e. to remove the low quality and non-human reads before the alignment to the reference genome. The strength of DecontaMiner is the flexibility of its use, coupled with a complete, easy to plug-in, and automatic pipeline.

## Methods

**Fig. 1:**
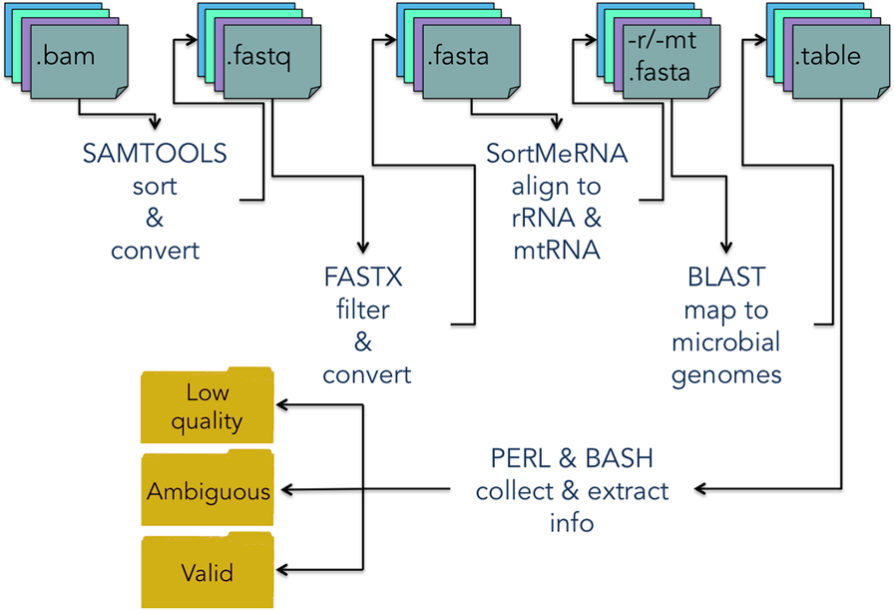
The DecontaMiner pipeline. The tools and the relative functions, input and output file formats are shown. The outputs are grouped in three main directories: ‘Low quality’, ‘Ambiguous’ and ‘Valid’, which collect the result files of each analyzed sample

DecontaMiner has been developed to work on one or more samples, and both on paired- and single-end experiments. The input is a directory containing all the samples to analyze. The tool is composed of two main modules, the first one involving the format conversion, filtering and mapping steps, and the second one performing the extraction and the parsing of the results. The first module automatically and sequentially executes all the steps up to the alignment to the microbial genomes. All the single scripts belonging to this module are provided, allowing the user to run them separately, depending on the needs and data. The second module is composed of two parts, one for the filtering of the BLAST alignment results, and one to collect the information accordingly to user defined settings. The code is written in Perl, with bash scripts to connect and launch the various submodules of the pipeline. A schematic view of the pipeline is shown in Fig. 1.

DecontaMiner has the following dependencies from external tools: Samtools [44], FastX toolkit [45], SortmeRNA [46] and BLAST [47]. These softwares are widely used by the NGS community. The input files are accepted in fastq, fasta and bam formats, and this option determines the starting point of the pipeline. If a bam file is given as input, it is converted first to the fastq format and then to fasta. The quality parameters to retain or discard reads can be set by the user to override default values. The sequences which pass the filtering step are then aligned against the human ribosomal and mitochondrial RNA using SortmeRNA, a software designed to this aim. For DNA sequencing data it is sufficient to omit this step.

The hypothetical non-human sequences can then be mapped to bacteria, fungi and viral genome databases (NCBI nt) using the MegaBLAST algorithm and specifying the alignment length and the number of allowed mismatches/gaps. The BLAST databases have been created downloading the sequences of the complete genomes from the RefSeq repository through the biomartr 0.7.0 R package [48]. The fasta files have then been assembled as blast databases trough the blast command “makeblastdb”. The databases are available for download at the DecontaMiner website. The user can also create its own db and provide it to the tool, simply indicating the absolute path in the configuration file.

The output from the BLAST alignment step is in tabular format and contains all the matches satisfying the alignment criteria. Additionally, the files containing the reads discarded along the overall pipeline are also generated. Hence, low quality, rRNA/mtRNA-mapped, ambiguous and unaligned reads are all stored, to allow users to trace every single unmapped read along the whole process. To execute the second module some thresholds must be specified. In particular, the user must indicate the match count threshold (MCT), i.e. the minimum number of total reads successfully mapped to a single organism to consider it as a contaminant. The default is five, a very low threshold, so that the user can have the whole list of the possibly significant detections. However it is highly recommended to tune this parameter based on the size of the database, and the aim of the performed analyses. The uniqueness of the mapping is evaluated at the genus level, i.e. considering ambiguous two reads perfectly mapping on different genera. The results are extracted and organised both by genus and species annotation.

The result files are collected and grouped in three main output directories: ’Low quality’, which contains, for each sample, reads not compliant with the filtering parameters (i.e. length of alignment, number of allowed gaps and mismatches); ’Ambiguous’, containing, for each sample, the reads list and the tables of the ambiguous reads (i.e. paired reads not aligning on the same organisms, or reads showing correct matches with more than one genus); ’Valid’, containing tables and match counts of the alignment satisfying the filtering and collecting criteria. Additionally, a matrix reporting the percentage of species detected above a specified count threshold is produced and stored in this directory. This matrix, containing the distribution of contaminating organisms in all the samples, can be used to easily create a bar plot or other desired charts. Besides, DecontaMiner builds an offline HTML page containing summary statistics and plots, for the overall experiment and each sample. The interactive plots are obtained using the D3 javascript libraries [49].

DecontaMiner code is freely available for download at the website http://www-labgtp.na.icar.cnr.it/decontaminer, together with a toy example and the user guide. Furthermore, it is possible to upload the matrix file into a dedicated area of the above cited DecontaMiner online website. This functionality allows users to filter the results and narrow the search of interesting contaminants by selecting a subset of samples and/or setting up thresholds for the contaminants abundance.

## Results

This section is organised as follows: the first part is a short comparison on synthetic data between DecontaMiner and other available tools, in which we discuss what are the features that make DecontaMiner the best choice to detect and analyze contamination in unmapped NGS data; the second part is devoted to the analysis of the biological results obtained by the tool on two different NGS datasets.

### Accuracy assessment and comparison with other tools

We decided to compare DecontaMiner against CaPSID [39], FastQ Screen [40], and TruePure [42], since they are, to the best of our knowledge, the only software performing a similar contamination check. Unfortunately, it has been impossible to successfully install CaPSID: the tool relies on very old versions of the underlying software, and seems as not maintained since 2012. In Table 1 the principal features of the three compared software are shown. It is worth noticing that DecontaMiner is the most flexible and complete tool: it allows for multiple samples processing at the same time, in several input formats; it process paired-end reads, enforcing the consistency of contamination detection (two mate pairs must align on the same organism to be counted as a match); it does not have any limitation on the number or size of input samples, and supports any kind of contaminating organisms database provided in blast index format. Additionally, Decontaminer gives the possibility to check the quality of the reads and filter them accordingly. When screening for contamination in a human samples, DecontaMiner performs a preprocessing step of mitochondrial and ribosomal reads removal, to avoid false detections due to the high number of copies of these RNAs and similarity of those sequences among different species. It is also possible to fine-tune the DecontaMiner stringency setting different parameters to filter the BLAST output according to the user demand. On viral genomes, for instance, it is important to allow gaps or mismatches, thus taking into account their high variability with respect to the reference. Another important feature of DecontaMiner is the possibility to have different views of the data: a coarse-grained one at the sample level, and a fine-grained view at the level of the single reads.

**Table 1:** DecontaMiner, TruePure and FastQScreen feature comparison

		DecontaMiner	TruePure	FastQScreen
Input type:	bam	$\checkmark $	×	×
	fastq	$\checkmark $	$\checkmark $	$\checkmark $
	fasta	$\checkmark $	$\checkmark $	×
	Multiple samples processing	$\checkmark $	×	×
	Paired end processing	$\checkmark $	×	×
	Unlimited input	$\checkmark $	×	×
	User defined databases	$\checkmark $	×	$\checkmark $
	Read tracking	$\checkmark $	×	×
	Parameter tuning	$\checkmark $	×	×
	Runs on HPC	$\checkmark $	×	$\checkmark $
	Visual output	$\checkmark $	$\checkmark $	$\checkmark $

To test the level of accuracy of DecontaMiner and to compare its performances with the other softwares, a synthetic sample was generated using the InSilicoSeq tool [50]. The test file contains reads coming from the human genes (≈77.5*%* of the total), from ten bacterial (≈18.1*%*), nine viral (≈4.1*%*) and four fungal (≈0.3*%*) genomes. About one million reads were generated, but, since only DecontaMiner supports paired-end processing, the file was split into two, and only half a million single-end reads were considered. Moreover, only DecontaMiner is able to process the whole file: TruePure manages no more than ten thousand reads, and FastQScreen one hundred thousand. Obtaining comparable results was not an easy task: TruePure and its provided extraction tool do not extract randomly the reads from the input file, but simply take the first ten thousand. Being the human reads at the beginning of our input file, no contamination at all was initially detected by TruePure. To evaluate the accuracy of the results, a five-thousand reads file containing the same fraction of genomes of the whole input was manually built and given in input to TruePure.

TruePure uses internal databases that can not be changed or updated, whereas DecontaMiner and FastQScreen were tested on the complete Bacteria/Fungi/Viruses databases obtained form NCBI. FastQScreen gives the possibility to choose among three different aligner (BWA, Bowtie, and Bowtie2) and requires the specific index files of the reference genomes, but it fails to manage the large indexes created by Bowtie and Bowtie2 in case of genomes greater than 4 billion nucleotides in length. The results are summarised in Table 2 and in Table 3. The comparison among the softwares was not straightforward, since FastQScreen does not provide information on the single species distribution, but only an overall result. In Table 2 the simulated data read counts and fraction on the non-human species are reported, alongside the results obtained by the three softwares. Although working on a manually curated input TruePure was not able to detect fungal contamination, while extracting correct percentages of bacterial and viral contamination. FastQScreen detects also the fungal contamination. Both FastQScreen and DecontaMiner detect lower percentages of fungal and viral contamination, and a higher bacterial one. Nonetheless, the results of DecontaMiner in terms of correctness are very high, as Table 3 shows. It is worth to note that DecontaMiner is able to give a very detailed species report while working on the complete databases and on a half million reads. TruePure results are biased, since the read in the input file were manually chosen to be representative of all the species. Nonetheless, the tool is not able to detect at all the fungal contamination, and misses one of the viruses. Details on the used data, and the obtained results are available in the Additional file 1.

**Table 2:** Sinthetic reads compared to DecontaMiner, TruePure and FastQScreen detected reads

	Simulated data		DecontaMiner		TruePure^a^		FastQScreen^b^	
	Read	%	Valid	%	Sequences	%	One hit/one	%
	counts		reads		found		genome	
Bacteria	99816	80.28	80527	92.73	975	80.25	13986	86.41
Fungi	2011	1.62	47	0.05	0	0	3	0.02
Viruses	22504	18.1	6263	7.22	240	19.75	2196	13.57
Total	124331	100	86837	100	1215	100	16185	100

^a^These are the results on the manually curated input file;

^b^For FastQScreen only the hits mapping on a single genome are shown

**Table 3:** Species detection: precision and recall for DecontaMiner, TruePure and FastQScreen

	DecontaMiner		TruePure^a^		FastQScreen^b^	
	Precision	Recall	Precision	Recall	Precision	Recall
Bacteria	0.82	0.9	0.67	1	NA	NA
Fungi	1	1	0	0	NA	NA
Viruses	0.92	1	0.53	0.89	NA	NA

^a^These are the results on the manually curated input file

^b^FastQScreen does not provide a detailed report on the distribution of the hits found

### Tests on biological data

The DecontaMiner pipeline has been tested on two publicly available datasets downloaded from the GEO (Gene Expression Omnibus) portal. These datasets have also been used to test the first prototype as described in [43]. However, the pipeline has changed since then, and the NCBI databases of contaminant organisms as well. The first dataset (GSE69240) contains 25 pure HG-DCIS (High-Grade Ductal Carcinoma In Situ) and ten normal breast organoids samples. RNA was polyA enriched, and 76 nt paired-end sequencing was performed. The second dataset (GSE68086) contains 228 samples plus two replicates of six different malignant tumors and 55 samples plus two replicates of healthy donors. Total RNA from blood platelets was sequenced in a single-end mode and with 101-bp reads. The data in SRA (Sequence Read Archive) format were downloaded and converted to fastq format using the SRAToolkit [51].

**Fig. 2:**
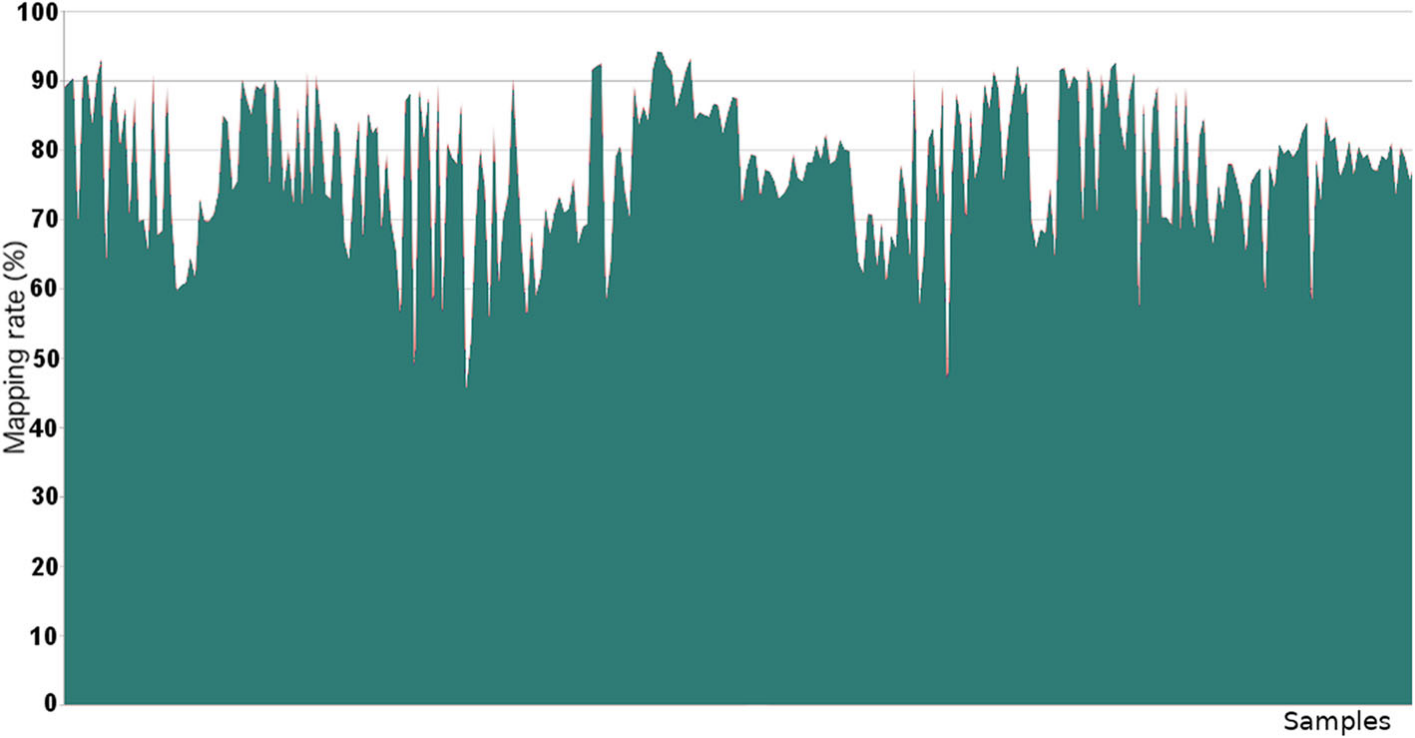
Overall read mapping rate distribution (GSE68086) Area chart showing the mapping rate of the GSE68086 dataset samples. The amount of mapped reads ranges from 45.5% to 94.2%, indicating a great variability among samples

**Fig. 3:**
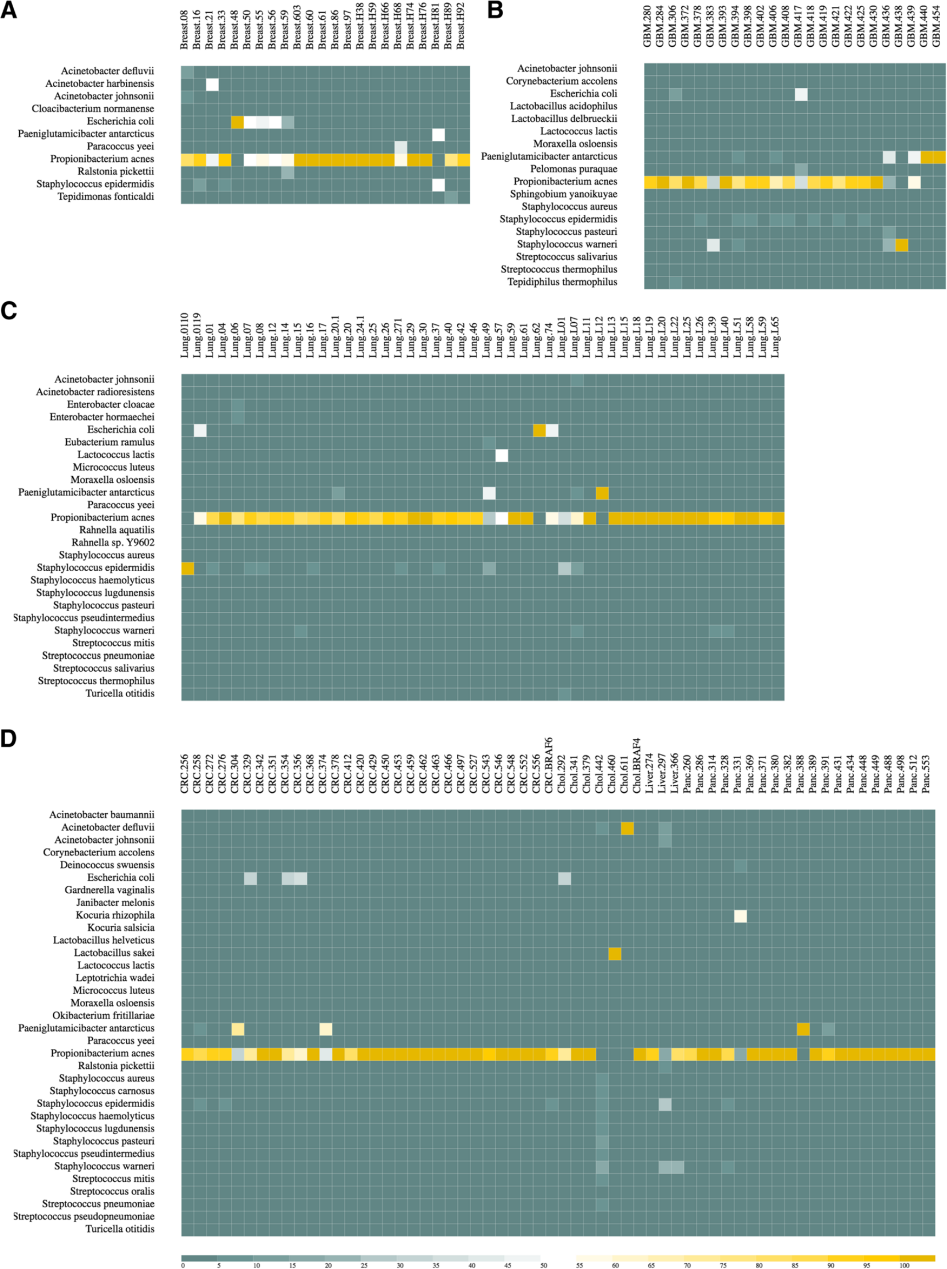
Bacterial abundance in the tumoral samples. The heatmaps show the relative abundance of bacterial species in 4 tumor types: breast cancer (**a**), GBM (**b**), Lung (**c**) and digestive system cancers (**d**). Bacteria with a match count ≥ 100 and a relative abundance ≥ 5% in at least one sample/group are shown. *P. Acnes* is highlighted by a dot in all groups, being the most abundant contaminant. The heatmaps are generated by the DecontaMiner offline HTML page and online website

The reads quality was assessed by FastQC [52]. FastQ files were aligned to the reference genome (assembly hg19) using the fast splice junction mapper TopHat [53] guided by UCSC gene annotation. The alignment statistics were checked by SamStat [54]. The reads which failed to map were stored in a separate bam file for each sample and put in the same directory, given as input to DecontaMiner. The samples from the dataset GSE69240 show a good and consistent mapping rate for all the samples, and, as expected, we did not observe matches to contaminating genomes. We also lowered the analysis stringency with respect to allowed mismatches and gaps (2 for each), obtaining the same outcome. This result totally agrees with the experiment characteristics. Moreover, to confirm DecontaMiner’s results, we processed the same samples with FastQScreen, obtaining the same outcome. Indeed, an efficient polyA enrichment and a sterile environment should guarantee contamination-free samples. The absence of matches may suggest a sterile working environment and careful experimental processes. The mapping rate of GSE68086 samples, instead, shows a high variability (Figs. 2 and 3).

The number of unmapped reads range from 5 to 40% and show several matches to microorganism genomes. Only contaminants having at least 100 matches were retained for further investigations (MCT = 100). Among them, we considered only organisms having a relative abundance ≥ 5% in at least one sample of the two groups. These settings were chosen to avoid weak detections, and to extract only contaminations significant across all the samples. A summary of the obtained results is reported in Table 4.

**Table 4:** Number of contaminating genera and species having at least one hundred matches, and a relative abundance ≥ 5% in at least one sample/group are shown, for each of the three considered kingdoms and for Tumor and Control samples

	Tumors		Controls	
	Genus	Species	Genus	Species
Bacteria	100	199	57	91
Fungi	12	15	8	10
Viruses	/	9	/	7

**Fig. 4:**
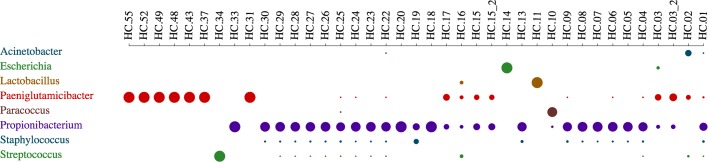
Bacterial abundance in the healthy samples. The dot chart shows the relative abundance of bacteria, grouped by genus. Only genera with a match count ≥ 100 and a relative abundance ≥ 5% in at least one sample are shown. The dot size is proportional to the abundance. The most relevant bacteria belong to the Paeniglutamicibacter and Propionibacterium genera. The dot charts are generated by the DecontaMiner offline html page and online website

It is evident that tumur samples show a higher number of detected microorganisms than the control samples. The quality parameters set to filter the BLAST alignments were very stringent: match length equal to the read length; no gaps; no mismatches. Collecting the results obtained from the alignment to bacterial genomes, we observed many matches to *Propionibacterium Acnes* in almost all samples, both from tumors and healthy donors, suggesting the possibility of either a downstream or a common blood platelets contamination (Figs. 3-4).

Indeed, as reported by literature, *P. acnes* is a ubiquitous bacterium and its presence has been detected in human tissues, hospital devices, lab reagents and environment [55]. Furthermore, bacterial contamination of blood components is one of the most challenging issues of transfusion medicine and sepsis [56, 57]. Bacteremia diagnosis can be performed by using NGS approaches [58, 59]. *Propionibacterium Acnes* is considered to be one of the most frequent contaminants of platelet concentrates [60, 61]. Except for the background contamination of *Propionibacterium Acnes*, some samples seem to be more contaminated than others, suggesting a different timing of sample processing, or an upstream rather than downstream contamination. In particular, these contaminations involve *E. coli* and several species of *Staphylococcus* and *Acinetobacter* genera. Multiple studies have demonstrated that pathogenic *E. coli* strains can be related particularly to gastrointestinal cancers, since these strains have the potential to transform enterocytes by cyclomodulin toxin effects and promote the development of cancer [62, 63]. Also almost all healthy control samples show a remarkable amount of *Propionibacterium* genus (Fig. 4), strengthening the hypothesis of a downstream contamination during sample processing. Along with it, also *Paeniglutamicibacter* is clearly present in some of the samples. Reclassification of some species of the genus *Arthrobacter* into novel genera, among which *Paeniglutamicibacter*, have been recently proposed [64]. The *Arthrobacter* genus belongs to the *Actinobacteria* phylum and is found primarily in soil.

**Fig. 5:**
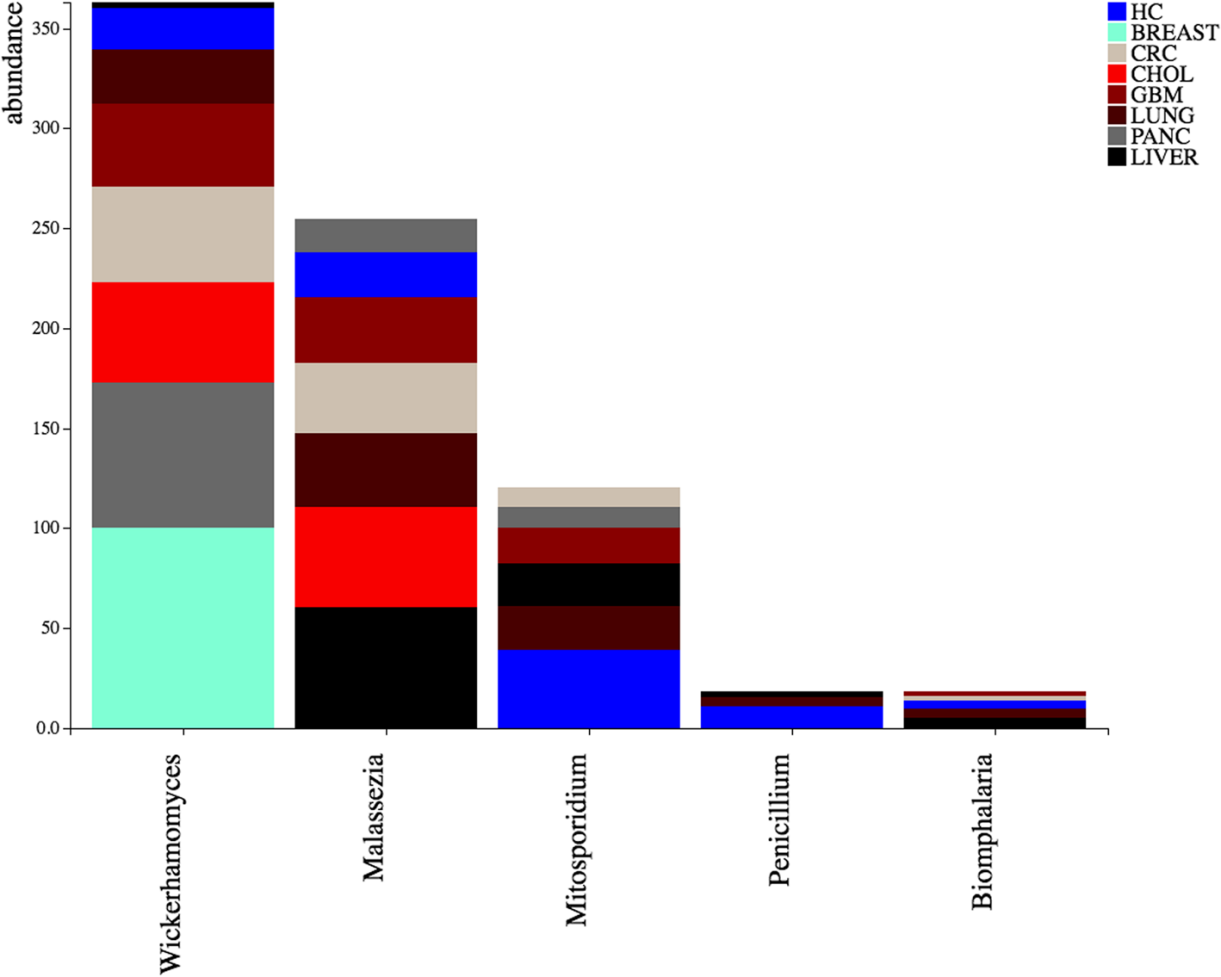
Fungi contamination in tumor and healthy groups. The stacked bar chart shows the fungal genera having an average value in all groups ≥ 10%, considering tumor and healthy (HC) groups. Bars are stacked by the group for which the contaminating organism (x-axis) has been detected. The y-axis scale reports the sum of the values in all the samples. Groups are ranked in a increasing order, in terms of contaminant abundance, from the bottom to the top. The stacked bar charts are automatically generated by the DecontaMiner online companion website

Compared to bacteria, a more modest amount of reads aligned to fungal genomes. Both tumor and healthy samples show, as most predominant species, fungi and yeasts that can be ascribed to environmental contamination (Fig. 5). In particular, *Wickerhamomyces* species are often recovered from arboreal habitats [65, 66]. *Malassezia* species are skin commensal and frequently found as laboratory reagent contaminants [67, 68]. *Penicillium*, mostly present in healthy samples, is a common air contaminant [69].

Concerning viruses, we predominantly found matches to *Enterobacteria phage*, and, to a lower extent, to *Propionibacterium phage* and *Staphylococcus phage*. This result can be considered a further confirmation of the detection of the bacterial species, since bacteriophages are commonly found where their bacterial hosts are present, including the human body [70], but also it suggests the presence of cloning vectors contamination. Another finding worth to be mentioned is the alignment of some samples to the human *Herpes virus*. Association of this particular virus with cancer and its feasible etiologic role in tumorigenesis have been largely studied [71, 72].

## Conclusions

DecontaMiner is a tool designed and developed to investigate the presence of contaminating sequences in NGS data. It analyzes the sequences rejected during the alignment to the reference genome, the so called *unmapped reads*. The sequences in input can be in fastq, fasta or bam format. Hence, DecontaMiner can be used both as a filtering tool, to remove foreign reads from the raw sequencing file, usually in fastq or fasta format, and as a detection tool to identify contaminating sequences among the unmapped reads, generally stored in a bam file.

The novelty of DecontaMiner is mainly represented by its easy integration with the standard procedures of NGS data analysis, thus making DecontaMiner a useful tool for additional investigation of the data and condition under study. We assessed the accuracy of DecontaMiner on a synthetic dataset that was also used to compare its performances with similar tools: the results show that DecontaMiner is both reliable and precise, while being highly flexible in the choice of databases and filtering parameters. To test the functionality of our tool on real data, we used two different RNA-Seq datasets. The lack of matches to microorganisms in the case of the polyA-RNA samples (GSE69240) was in perfect agreement with the nature of the experiment. The reliability of our pipeline was further tested on a dataset of total RNA sequencing (GSE68086) of tumor and healthy samples. We found in almost all the samples a background contamination of *P. Acnes*, which is very well known as common contaminant of hospital and laboratory environments. From the alignment to fungal and virus genomes the matches were very modest compared to bacteria, although the mapping to bacteriophages was in agreement with what we found as bacterial contamination.

In conclusion, DecontaMiner can suggest the presence of contaminating organisms in samples sequenced by NGS, that might derive either from laboratory contamination or be part of their biological source, and can be considered as worthy of further investigation and experimental validation.

## Additional file

Additional file 1 This Excel file includes several sheets containing all the details on the data used for the accuracy assessment and the comparison with other tools, and the obtained results. (XLSX 139 kb)

## Abbreviations

BAM: Binary alignment map
BLAST: Basic local alignment search tool
mtRNA: mitochondrial RNA
nr: non-redundant
nt: nucleotides
NCBI: National center for biotechnology information
NGS: Next generation sequencing
polyA: polyadenylated
RNA-seq: RNA sequencing
rRNA: ribosomal RNA
